# Adolescent idiopathic scoliosis and spinal fusion do not substantially impact on postural balance

**DOI:** 10.1186/s13013-015-0042-y

**Published:** 2015-06-09

**Authors:** Janneke JP Schimmel, Brenda E Groen, Vivian Weerdesteyn, Marinus de Kleuver

**Affiliations:** Sint Maartenskliniek Research, Sint Maartenskliniek, P.O box 9011, Nijmegen, GM The Netherlands 6500; Department of Orthopaedic Surgery, Sint Maartenskliniek, Nijmegen, The Netherlands; Department of Orthopaedic Surgery, VU university medical centre, Amsterdam, The Netherlands; Radboud University Medical Centre, Donders Institute for Neuroscience, Department of Rehabilitation, Nijmegen, The Netherlands

**Keywords:** Adolescent idiopathic scoliosis, Postural balance, Spinal fusion

## Abstract

**Background:**

The spinal curvature in patients with Adolescent Idiopathic Scoliosis (AIS) causes an asymmetry of upper body postural alignment, which might affect postural balance. However, the currently available studies on balance in AIS patients are not consistent. Furthermore, it is not known whether potential deficits are similar between patients with single and double curves. Finally, the effects of a corrective posterior spinal fusion on postural balance have not yet been well established.

**Methods:**

Postural balance was tested on a force plate, in 26 female subjects with AIS (12–18 years old; preoperative Cobb-angle: 42-71°; single curve n = 18, double curve n = 6) preoperatively, at 3 months and 1 year postoperatively. We also conducted a balance assessment in 18 healthy age-matched female subjects. Subjects were tested during quiet double-leg standing in four conditions (eyes open/closed; foam/solid surface), while standing on one leg, while performing a dynamic balance (weight shifting) task and while performing a reaching task in four directions.

**Results:**

AIS subjects did not demonstrate greater COP velocities than controls during the double-leg standing tasks. In the reaching task, however, they achieved smaller COP displacements than healthy controls, except in the anterior direction. AIS patients with double curves had significantly greater COP velocities in all test conditions compared to those with a single curve (p < 0.05). For the AIS group, a slight increase in COP velocities was observed in the foam eyes closed and right leg standing condition at 3 months post surgery. At 1-year post surgery, however, there were no significant differences in any of the outcome measures compared to the pre-surgery assessment, irrespective of the curve type.

**Conclusions:**

Postural balance in AIS patients scheduled for surgery was similar to healthy age matched controls, except for a poorer reaching capacity. The latter finding may be related to their reduced range of motion of the spine. Patients with double curves demonstrated poorer balance than those with a single curve, despite the fact that they have a more symmetrical trunk posture. Postural balance one year after surgery did not improve as a result of the better spinal alignment, neither did the reduced range of trunk motion inherent to fusion negatively affect postural balance.

## Background

Scoliosis is the most common type of spinal deformity during adolescence and is defined as a lateral curvature of the normally straight spine. The curvature is the product of an axial rotation and subsequent decreased kyphosis of the thoracic spine. For most patients the cause of scoliosis is not known and it is therefore diagnosed as adolescent idiopathic scoliosis [1]. There has been a lot of speculation concerning the etiology of AIS, and some studies have focused on possible disturbed motor-sensory integration.

The scoliotic curvature causes an asymmetry of upper body postural alignment, and this might affect postural balance. However, the currently available studies on balance in scoliotic patients are not consistent. Some studies showed that AIS patients do not have impaired postural balance when compared to healthy controls [2–5], while several others did find an effect of AIS on postural balance [6–14]. This discrepancy in findings may be due to differences in curve characteristics included and their effects on postural balance. The included curve types (single or double), number of different curve types, location of curves (thoracic and lumbar), and/or Cobb angles vary considerably between studies, and all of these factors individually have been shown to influence postural balance. For example, patients with single lumbar curves showed poorer postural balance than those with double major curves [15] and greater Cobb angles were also associated with poorer postural balance [7, 16].

Furthermore, the discrepant results may be due to the use of different study methodology. Different experimental tasks (static and/or dynamic posturography ([8, 12, 16] vs [4, 15, 16]) and conditions (e.g. with or without sensory deprivation; [3, 12–14] vs [6, 9]) have been studied with a great variety of outcome measures, which hampers a direct comparison between studies. Therefore, a thorough comparison of static and dynamic postural balance under various conditions of sensory deprivation/manipulation between a group of AIS subjects and age-matched healthy controls in a single study would be of great interest to determine if AIS is related to disturbed postural balance.

Another important question regarding postural balance in AIS patients relates to effects of treatment. In patients with Cobb angles >40-50 degrees, spinal fusion is the recommended treatment. It (partly) restores postural alignment of the trunk, which may be beneficial to postural balance. On the other hand, the resulting reduction in spinal mobility may be detrimental. Only a few studies have been published on the effects of this surgical procedure on postural balance, but the inconsistencies in the results preclude drawing firm conclusions [5, 17, 18]. Hence, the impact of corrective spinal fusion on postural balance) is still not sufficiently clear.

The purpose of this study was to determine whether AIS patients have defective postural balance compared to a healthy age-matched control group and whether potential deficits are similar between patients with single and double curves. The second purpose was to delineate the effects of corrective posterior spinal fusion on postural balance in the same group of patients with AIS. We hypothesized that postural balance in AIS patients before and after surgery is poorer than in healthy age-matched controls, patients with double curves have better postural balance than those with single curves due to more balanced symmetry of their trunk. Furthermore, we expected AIS patients one year after surgery to have improved postural balance compared to their pre-operative performance due to the restoration of postural alignment of the trunk.

## Methods

### Participants

The AIS population was recruited from the patients scheduled for a posterior spinal fusion, who presented at the Department of Orthopaedics between January 2009 and December 2011. Curve types were classified according to the Lenke classification: patients with main thoracic curves who underwent selective thoracic fusion (Lenke 1a, 1b, 2a; so-called ‘single curves’) and patients with a thoracic and lumbar component who underwent thoracic and lumbar fusion (Lenke 1c, 2b, 3c, 4c, 6; so-called ‘double curves’). There were no other curve types in this cohort. All patients were treated by a specialized orthopaedic spine surgeon. Posterior instrumented spinal fusion was performed with predominantly pedicle screw based instrumentation. The surgeries were performed under motor evoked potential spinal cord monitoring. All patients received the standard hospital pre-operative, intra-operative and postoperative care.

The inclusion criteria for the present study were diagnosis of AIS; female gender; and age between 12 and 18 years. Patients were excluded if they had a history of spine surgery, mental retardation or other musculoskeletal/neurological diseases known to affect sensorimotor performance. The researcher contacted eligible participants by telephone and provided them with oral information supplemented by an information sheet. The control group was recruited from the local Secondary Education school and consisted of 18 healthy female subjects with no diagnosis of scoliosis or any other musculoskeletal or neurological problems.

After approval of the hospitals’ investigational review board and the local medical ethics committee (Independent Review Board Nijmegen, IRBN) and Reade METC, the study was conducted at the Sint Maartenskliniek Nijmegen, The Netherlands. Written informed consent was obtained from all participants and their parent(s) or guardian(s).

### Experimental setup and protocol

The AIS patients underwent three balance assessments: before surgery (T0), at three months (T1) and one year after surgery (T2). The control group was evaluated once.

Measurements of quiet standing and dynamic posturography were conducted on two force platforms [19]. Each force platform was mounted on three force transducers, which recorded the vertical ground reaction forces. Safety bars were mounted next to the platforms to prevent falls. The participants stood barefoot on the platforms with their arms by their sides while facing a computer screen. The position of the feet was standardized by a fixed frame mounted on the platform (medial sides of the heels 8.4 cm apart and the feet toeing-out at 9°).

For the quiet standing tasks, subjects were asked to stand as still as possible. After a five-second countdown, an auditory signal sounded the start of each task. Six quiet standing tasks were carried out in the following order: standing on a solid surface with eyes open (SEO), standing on a solid surface with eyes closed (SEC), one-legged standing with eyes open on solid surface first left (LSEO), then the right leg (RSEO), standing on a foam surface with eyes open (FEO) and standing on a foam surface with eyes closed (FEC). Each task was tested twice in counterbalanced order. For the eyes closed conditions, subjects were blindfolded with a pair of dark goggles. For the trials on a foam surface, subjects stood on 45 mm thick foam providing a compliant surface. Trial duration was 30 seconds, except for the one-legged standing tasks which lasted 15 seconds.

Secondly, a dynamic balance task was performed, which involved 30-seconds of weight-shifting [20, 21]. Subjects were instructed to move their weight from the left leg to the right leg and vice versa. Their Centre of Pressure (COP) position was visualized as a moving dot on a computer monitor in front of the them, with two boxes representing the target areas. Once the cursor was held in the box for one second, the other box changed color and the subjects had to switch their weight to the other leg. After 10 seconds of practice, the task was performed twice.

Finally, a reaching task was performed in four directions. Subjects were encouraged to reach as far as possible in the anterior, posterior, left and right direction, without lifting their feet from the force platform. After they had reached the maximal position, they were asked to maintain this position for 15 seconds and the measurement was started. Each direction was tested twice, both series in the same order.

### Data-analysis

Details on the computational analysis of the data processing were presented in several previous papers [19, 20, 22]. The signals from the force transducers were used to calculate the point of application of the COP in the two-dimensional transverse plane. The maximum error in the mediolateral and anteroposterior direction was ±1 mm [22]. The lag time between movement of the COP and the square was about 16 ms [20].

The subject’s Base of Support (BoS) was measured in a purely anteroposterior (AP) and mediolateral (ML) direction. For the reaching task the outcome measure was the position of the COP. The position of the COP in the AP direction was expressed in as a percentage of the BoS length measured from the back of the subjects heels (COPy). In the ML direction, it was expressed as a percentage of half the BoS width measured from the midline of the force platform (COPx). For the quiet standing tasks, COP excursions were quantified as the root mean square (RMS) of the velocity (VCP), for the AP and ML directions separately (respectively VCPx and VCPy). To evaluate the effects of visual deprivation on quiet standing balance, Romberg’s quotient was calculated for both solid and foam surface as the VCP in the EC-condition divided by the EO-condition (SEC/SEO and FEC/FEO, respectively) in both the AP and ML-direction.

In the dynamic balance task, the number of weight shifts was counted (score) and the movement time in both left-right and right-left directions were calculated. As these outcomes did not yield asymmetric results, we included the mean movement time in our analyses.

### Statistical analysis

The averages of the two repetitions of each of the balance tasks were used for analysis. Student T-test’s were used to evaluate differences in preoperative values (T0) between the AIS and the control group.

To determine the effect of surgery and curve type, a repeated measures ANOVAs was performed with *Time* as within-subjects variable and *Curve type* (single vs double) as a between-subjects variable for each task. The interaction effect (*Curve type* x *Time*) was incorporated in the model and post-hoc t-tests with Bonferroni correction were performed. All analyses were performed with the statistical package of SPSS 20 (IBM SPSS Statistics, version 20), with a significance level of p < 0.05.

## Results

A total of 26 patients were included in this study (Table 1). The curves of eight AIS patients were classified as double curves and the remaining 18 as single curves. Two patients (ID 17 and 22) were assessed pre-operatively, but declined further participation in the study before the first post-operative measurement, and therefore these subjects were excluded from the analyses. One patient (ID 2) declined the 1-year post-operative measurement because of time constraints. Data of this patient was imputed by carrying the last observation forward (i.e. no progression occurred). One patient (ID 16) underwent an additional surgery shortly after the three-months post-operative measurement (extension of fusion from L3 up to L4). She had completely recovered by the time of the one-year follow-up measurement and therefore data of all the three measurements were included for the analyses. Hence, data of 24 patients (18 single curves and six double curves) were used for analysis. The age (mean ± SD) of these 24 AIS patients was 14.5 ± 1.7 years at the time of surgery. Pre-operative Cobb-angles ranged between 36 and 71°. They had a height of 161 ± 7.1 cm, weight of 49.2 ± 8.4 kg, and BMI of 18.8 ± 2.5 kg/m^2^. The group of healthy controls consisted of 18 girls with a mean age of 14.1 ± 1.2 years, height of 169 ± 6.1 cm, weight of 54.4 ± 6.5 kg, and BMI of 18.9 ± 1.5 kg/m^2^.

**Table 1:** Demographics and preoperative curve characteristics of AIS patients

ID	Age at surgery (years)	Pre-operative curve characteristics			Spinal fusion (number of fused vertebrae)
		Cobb-angle thoracic curve (°)	Lenke-classification	Single (S) / Double(D) curve	
1	15.4	47	6	D	T4-L3 (12)
2**	12.7	53	1C	D	T4-L4 (13)
3	12.7	62	1A	S	T5-L1 (9)
4	14.8	54	1A	S	T5-T12 (8)
5	15.8	54	1A	S	T5-T12 (8)
6	11.9	61	1A	S	T5-L1 (9)
7	15.2	52	3C	D	T4-L4 (13)
8	16.1	36	1A	S	T5-T12 (8)
9	12.0	46	1B	S	T4-T12 (9)
10	12.3	55	1A	S	T5-T12 (8)
11	14.7	58	1B	S	T4-T10 (7)
12	14.9	70	3C	D	T3-L4 (14)
13	15.4	54	1A	S	T4-T12 (9)
14	15.6	52	2A	S	T6-L2 (9)
15	15.2	50	1B	S	T5-T12 (8)
16•	18.5	50	3C	D	T5-L4 (12)
17*	16.1	54	3C	D	T4-L3 (12)
18	12.6	71	2A	S	T3-L1 (11)
19	16.5	45	1B	S	T5-T12 (8)
20	13.7	44	1A	S	T5-L2 (10)
21	12.5	58	1A	S	T5-T12 (8)
22*	14.7	49	4C	D	T4-L3 (12)
23	12.3	52	1B	S	T5-L1 (9)
24	14.7	64	3C	D	T5-L3 (11)
25	15.9	42	2A	S	T3-L2 (12)
26	15.5	55	1A	S	T5-L1 (9)

*Lost-to-follow before second measurement

**missed final measurement

•underwent additional surgery after 3 months (extension of fusion from L3 up to L4)

### Comparison between groups at T0

For the one-leg as well as double-leg quiet standing tasks, the analysis yielded no statistical differences between the control and AIS group at T0 for any of the conditions and outcome measures (Table 2). Consequently, Romberg’s quotient neither revealed differences between groups (Table 2).

**Table 2:** Mean (±SD) of double-leg quiet standing tasks

Outcome measure VCP (mm/s)	Control group (n = 18)	Single curve (n = 18)			Double curve (n = 6)		
		*Pre-operative*	*3 months*	*1 year*	*Pre-operative*	*3 months*	*1 year*
SEO x	7.1 (2.8)	6.7 (3.5)	18.4 (2.6)	6.0 (2.7)	7.6 (1.3)	9.1 (2.7)	8.3 (2.3)
SEO y	8.7 (2.4)	8.8 (2.7)	9.2 (3.3)	8.6 (3.2)	11.6 (5.5)	12.3 (3.1)	11.9 (5.3)
SEC x^#^	9.8 (3.8)	8.6 (3.2)	8.6 (3.6)	8.8 (5.4)	11.7 (3.0)	13.7 (2.9)	12.2 (4.4)
SEC y^#^	13.5 (4.1)	13.1 (3.9)	13.0 (4.9)	13.3 (4.0)	17.4 (7.3)	18.7 (5.1)	18.2 (6.8)
FEO x^#^	9.5 (3.0)	8.5 (3.0)	8.5 (3.3)	7.9 (2.6)	11.3 (4.3)	12.1 (2.6)	11.0 (3.1)
FEO y^#^	13.6 (3.6)	10.8 (2.5)	11.7 (2.8)	12.1 (4.2)	15.0 (5.7)	15.3 (2.2)	13.7 (4.1)
FEC x^#^	16.6 (5.7)	15.1 (6.0)	15.5 (5.4)	15.6 (6.2)	20.5 (5.1)	24.1 (8.5)	20.8 (10.6)
FEC y^*,#^	27.1 (8.5)	22.6 (5.9)	24.3 (6.5)	23.7 (6.9)	30.7 (8.3)	36.2 (8.6)	30.3 (12.5)
RQ x Solid	1.4 (0.3)	1.4 (0.4)	1.3 (0.2)	1.4 (0.4)	1.5 (0.2)	1.6 (0.3)	1.5 (0.3)
RQ x Foam	1.8 (0.4)	1.8 (0.4)	1.9 (0.4)	2.0 (0.4)	1.9 (0.5)	2.0 (0.5)	1.8 (0.5)
RQ y Solid	1.6 (0.3)	1.5 (0.3)	1.4 (0.2)	1.6 (0.3)	1.5 (0.1)	1.5 (0.1)	1.6 (0.3)
RQ y Foam	2.0 (0.4)	2.1 (0.4)	2.1 (0.4)	2.0 (0.3)	2.1 (0.5)	2.4 (0.6)	2.2 (0.7)
LSEO x	29.5 (10.1)	25.2 (6.7)	25.1 (8.9)	25.2 (8.3)	33.1 (12.1)	35.0 (8.0)	30.6 (9.6)
LSEO y	31.1 (11.1)	27.2 (8.2)	27.2 (8.3)	25.4 (8.8)	37.5 (8.6)	35.1 (4.2)	30.6 (6.9)
RSEO x	27.2 (6.1)	25.5 (8.7)	24.6 (9.3)	23.6 (6.2)	34.8 (6.7)	33.9 (7.0)	30.4 (5.8)
RSEO y	27.3 (6.2)	26.8 (7.6)	26.8 (8.2)	26.0 (8.6)	37.8 (8.5)	37.4 (6.2)	34.0 (5.0)

RQ = Romberg’s quotient

*significant effect of *Time*

^**#**^significant effect of *Curve Type*

In the dynamic balance task no between-group differences were found in score (p = 0.89) or movement time (p = 0.50) (Table 3). For the reaching task, the T-tests showed significantly smaller average COP displacements (maintained over 15 seconds) in the posterior (p = 0.006), left (p = 0.009) and right (p < 0.001) directions in the AIS compared to the control group (Table 3).

**Table 3:** Mean (±SD) of reaching task, dynamic balance task

Outcome measure	Control group (n=18)	Single curve (n=18)			Double curve (n=6)			
		*Pre-operative*	*3 months*	*1 year*	*Pre-operative*	*3 months*	*1 year*	*p-value* ^*$*^
**Reaching task**								
COP position (%) Anterior	71.8 (9.5)	66.5 (9.5)	69.4 (10.0)	67.4 (10.6)	68.9 (5.3)	68.6 (7.6)	72.8 (7.1)	0.10
COP position (%) Posterior*	22.1 (6.2)	33.0 (14.3)	30.7 (16.4)	32.4 (18.3)	25.3 (7.3)	22.0 (8.9)	22.6 (3.8)	*0.006*
COP position (%) Left*	61.9 (9.8)	52.6 (11.5)	53.0 (13.8)	49.5 (13.1)	55.2 (6.4)	59.5 (9.3)	59.1 (5.8)	*0.009*
COP position (%) Right*	61.4 (9.4)	49.7 (8.6)	49.0 (17.2)	49.3 (12.9)	52.8 (8.7)	56.4 (7.9)	48.0 (22.7)	*<0.001*
**Dynamic balance task**								
Score	13.0 (1.6)	13.1 (2.9)	14.4 (3.0)	14.5 (3.0)	12.2 (4.3)	12.2 (2.2)	13.8 (3.7)	0.89
Movement time (s)	2.3 (0.3)	2.3 (0.4)	2.1 (0.4)	2.1 (0.5)	2.7 (1.4)	2.5 (0.5)	2.3 (0.7)	0.50

*significant different between control group and AIS patients at T0

*$* student t-test between control group and AIS patients at T0

### Effect of time

**Fig. 1:**
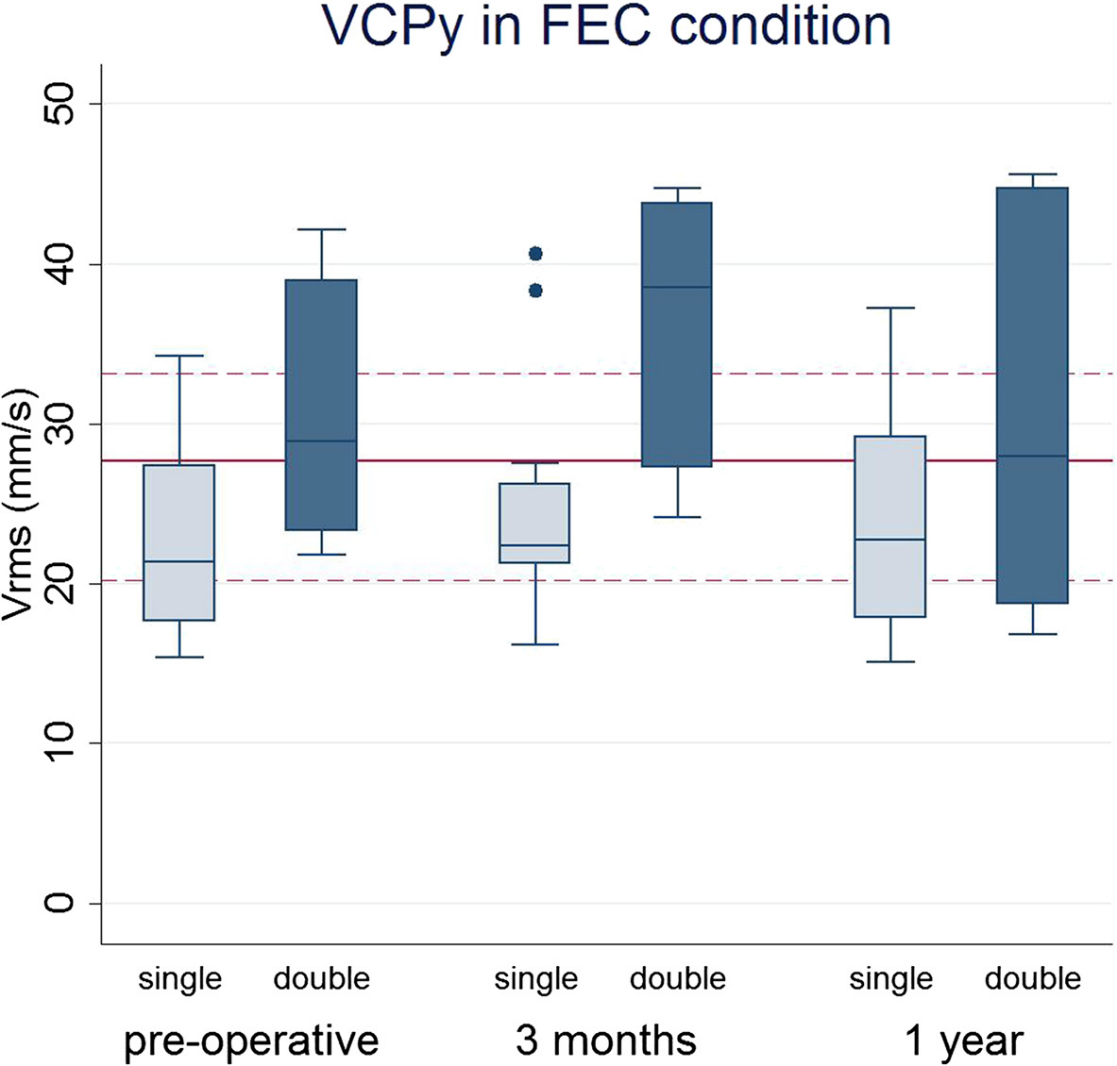
Velocity of the COP in the anteroposterior direction (VCPy) at the three measurements for patients with a single (light blue) and double (dark blue) curvature in the foam surface, eyes closed (FEC) condition. Red lines represent median value (with 25th and 75th percentile) of healthy control group. No differences were observed between the control group and AIS patients. Patients with double curves demonstrated significantly greater velocities (p < 0.05)

For the double-leg standing tasks, the repeated measures ANOVA showed a significant main effect of *Time* for VCPy in the FEC condition (F_2,44_ = 2.88, p = 0.02; Table 2, Fig. 1). Post-hoc analysis showed a significantly greater velocity at T1 compared to T0 (p = 0.025). For the other outcome measures, no main effects of *Time* were found. In addition, no significant *Time x* C*urve type* interactions were observed in any of the tasks.

For the LSEO task, a significant main effect of *Time* was observed for VCPy (F_2,44_ = 6.01, p = 0.005). Post hoc analysis showed significantly greater velocities at T0 and T1 compared to T2 (p = 0.002 and p = 0.008, respectively). For the RSEO task, no significant effect of *Time* was found (p = 0.315).

With respect to the dynamic balance and reaching tasks, no significant main effects of *Time* or *Time x* C*urve type* interactions were found (Table 3).

### Effect of curve type

In all double-leg quiet standing tasks except SEO, the repeated measures ANOVA showed a significant main effect of C*urve type* on VCPx and VCPy (p < 0.05), with the double curve group demonstrating greater velocities compared to patients with single curves (Fig. 1). Similarly, patients with double curves had greater COP velocities when standing on one leg as well (LSEO: F_1,22_ = 4.30, p = 0.050 for VCPx and F_1,22_ = 4.98, p = 0.036 for VCPy; RSEO: F_1,22_ = 6.56, p = 0.018 for VCPx and F_1,22_ = 9.8, p = 0.005 for VCPy).

No significant main effects of C*urve type* were found in Romberg’s quotient for the double-leg standing tasks, and for the outcomes of the dynamic balance and reaching tasks (Tables 2 and 3).

## Discussion

In a homogeneous group of surgically-treated AIS patients, we evaluated postural balance (1) compared with an healthy age-matched control group and (2) evaluated the effects of curve type and (3) the effects of a corrective posterior spinal fusion on postural balance. The results showed that in this cohort AIS patients in general do not have poorer postural balance compared to healthy age matched controls. Patients with double curves, however, had poorer postural balance than those with single curves. Compared to pre-operative values, a few outcomes of postural balance were slightly poorer three months post surgery, but had recovered one year after surgery. These results demonstrate the absence of marked improvement or deterioration of postural balance following spinal fusion in AIS patients.

In contrast to our hypothesis we did not find clear differences between AIS patients and healthy age-matched controls in the static and dynamic postural balance tests. The only task that did reveal differences between AIS patients and controls was the reaching task, with AIS patients achieving smaller COP displacements compared to controls. Overall, our findings were in agreement with the results of several other studies in which no differences between AIS and healthy controls were found in quiet standing postural balance [2–5]. Some other studies, however, did find poorer postural balance in AIS patients [6–14]. A direct comparison between studies is rather difficult, since a variety of study methodologies have been used with, consequently, a myriad of outcome measures. The discrepancies between study results may be due to differences in age and/or curve characteristics of the studied population. For example, age ranged from an average of 10 years [5] up to 16.5 years [14], and study populations included both right- and left-sided curves [12] as well as smaller curves (Cobb angle <30°) [6, 9, 16] compared to our studied population. A significant effect of curve type on postural balance was indeed demonstrated in the present study.

AIS patients with double curves showed poorer balance than those with single curves in the double and one leg standing tasks, while no differences were seen in the dynamic tasks and in reaching capacities. These findings do not support our hypothesis that the more symmetric trunk posture in patients with double curves would result in better postural balance compared to the a single right-sided curve type (that presumably shifts the center of mass of the trunk to the right). The presently observed effects of curve type on postural control are in contrast to those reported by Gauchard [15], who demonstrated that patients with double major curves performed better in both static and dynamic postural tests than those with single curves. The discrepancy may be due to differences in population characteristics, as Gauchard included both patients scheduled for surgery as well as those to be set in plaster or brace who, consequently, had smaller Cobb angles [15]. The use of a heterogeneous scoliosis group who are at different periods of progression may have influenced these results, since the Cobb angle itself has an effect on postural balance [7, 23]. Due to the large number of curve variations (e.g. Lenke type, Curve magnitude, curve flexibility), it is not possible to standardize the level or length of the spinal fusion in a patient cohort. We selected only AIS patients with right-sided curves scheduled for posterior spinal fusion, in whom we only distinguished between single and double curves based on the Lenke classification and not on the location of the apex of the spinal curve as in Gauchard’s study [15]. More strict inclusion criteria would diminish the generalisability of our study. It is yet unclear which underlying mechanisms may be responsible for the poorer balance in patients with double curves, which also persisted after surgery.

In contrast to our hypothesis, we did not observe substantial improvements of postural balance one year after corrective spinal fusion, despite marked improvement in trunk alignment. Importantly, we neither found signs of deterioration, including reaching capacities, despite the loss of spinal motion that occurs following fusion [24, 25]. The effects of surgery on postural balance have previously been reported in a few studies with different methodologies [5], study populations [17, 18] and varying follow-up time [5, 18] compared to our study [5, 17, 18]. Abreu et al. found that postural balance in 15 AIS patients (Lenke 3B and 3C) worsened immediately after surgery and remained inferior to preoperative values up to 90 days after surgery [18], which is in line with the deterioration we found in the FEC condition at three months. Also in line with our results at one year, no differences in clinical balance tests (i.e. Romberg test) were observed between pre-operative and six months post-operative balance assessments in a small group of AIS patients (n = 7) [5]. We focused primarily on the effects of surgery at one year, since clinically one would expect a complete recovery and solid fusion at that time. We are aware of only one study that reported postural balance data at one year follow-up, which results were inconsistent [17]. Some variables deteriorated compared to pre-operative values (e.g. mean sway distance), whereas other variables slightly improved (e.g. mean sway velocity and sway area) [17]. Hence, it appears that the measurement techniques used in our study, as well as in previous studies, may not be sensitive enough to detect potential subtle surgery-related differences in postural balance. More challenging tasks may be needed to identify such differences, yet it may also be questioned whether these would be clinically relevant. Probably, gait analyses using a three-dimensional motion analysis system may provide new information regarding the dynamic postural balance in addition to the outcomes of the semi-static tests on the force plates.

In this study, both adolescent AIS patients and age matched healthy controls demonstrated poorer postural balance than a historic adult control group measured on the same experimental setup in our institution [26]. For example, mean VCPy on solid surface with eyes closed (SEC condition) in healthy adults was 10.3 (±4.2) mm/s, compared to 13.5 (±4.1) mm/s in our control group and 14.2 (±5.1) mm/s in the AIS group [26]. These results suggest that age is a more important determinant of postural balance than the presence of a scoliosis. Indeed, it is known that during adolescence vision plays a predominant role in control of orientation and body stabilization, while proprioceptive/somatosensory information is under-used compared to adults [27, 28]. Hence, our findings also support that the suggestion that disturbed sensori-motor integration does not play a role in the development of the pathological spinal deformity [29]. Nevertheless, it is possible that the underuse of proprioceptive information at adolescent age masks postural control deficits, and that postural balance impairments problems might manifest themselves at adult age.

## Conclusion

Postural balance in the current cohort of 26 AIS patients with large curves is the same as in an age matched healthy control group. After surgery, in which a substantial part of the spine is made rigid by spinal fusion, some variables slightly deteriorated for a short period, but after one year, the patients’ postural balance had recovered to preoperative values. This suggests that disturbed motor-sensory integration does not play a role in the development of a spinal malalignment in AIS. These findings also suggest that postural balance in AIS is neither affected by the trunk asymmetry, nor by spinal fusion.

### Ethical review committee statement

The study was performed in accordance with the ethical standards in the Declaration of Helsinki and approved by the hospitals’ investigational review board and the IRBN. A waiver was received from the Reade METC for collection of data from the control group.

The work was performed at the Sint Maartenskliniek Nijmegen, The Netherlands.
